# On the importance of targeting parasite stem cells in anti-echinococcosis drug development[Fn FN1]

**DOI:** 10.1051/parasite/2014070

**Published:** 2014-12-22

**Authors:** Klaus Brehm, Uriel Koziol

**Affiliations:** 1 Institute of Hygiene and Microbiology, University of Würzburg Würzburg Germany; 2 Sección Bioquímica, Facultad de Ciencias, Universidad de la República Montevideo Uruguay

**Keywords:** Genome, Chemotherapy, Beta-tubulin, Benzimidazole, Stem cells, Germinative cells

## Abstract

The life-threatening diseases alveolar and cystic echinococcoses are caused by larvae of the tapeworms *Echinococcus multilocularis* and *E. granulosus*, respectively. In both cases, intermediate hosts, such as humans, are infected by oral uptake of oncosphere larvae, followed by asexual multiplication and almost unrestricted growth of the metacestode within host organs. Besides surgery, echinococcosis treatment relies on benzimidazole-based chemotherapy, directed against parasite beta-tubulin. However, since beta-tubulins are highly similar between cestodes and humans, benzimidazoles can only be applied at parasitostatic doses and are associated with adverse side effects. Mostly aiming at identifying alternative drug targets, the nuclear genome sequences of *E. multilocularis* and *E. granulosus* have recently been characterized, revealing a large number of druggable targets that are expressed by the metacestode. Furthermore, recent cell biological investigations have demonstrated that *E. multilocularis* employs pluripotent stem cells, called germinative cells, which are the only parasite cells capable of proliferation and which give rise to all differentiated cells. Hence, the germinative cells are the crucial cell type mediating proliferation of *E. multilocularis*, and most likely also *E. granulosus*, within host organs and should also be responsible for parasite recurrence upon discontinuation of chemotherapy. Interestingly, recent investigations have also indicated that germinative cells might be less sensitive to chemotherapy because they express a beta-tubulin isoform with limited affinity to benzimidazoles. In this article, we briefly review the recent findings concerning *Echinococcus* genomics and stem cell research and propose that future research into anti-echinococcosis drugs should also focus on the parasite’s stem cell population.

## Abstract (fr)

Les échinococcoses alvéolaire et kystique, deux maladies potentiellement mortelles, sont respectivement causées par les larves des vers plats *Echinococcus multilocularis* et *E. granulosus*. Dans les deux cas, les hôtes intermédiaires, comme l’homme, s’infectent par l’ingestion des oncosphères, suivie de la multiplication asexuée et la croissance presque illimitée du métacestode dans les organes de l’hôte. À côté de la chirurgie, le traitement des échinococcoses repose sur une chimiothérapie par les benzimidazoles, dont l’action est dirigée contre la bêta-tubuline du parasite. Cependant, comme les bêta-tubulines sont extrêmement similaires chez les cestodes et les humains, les benzimidazoles ne peuvent être utilisés qu’à des posologies parasitostatiques et sont associés à des effets secondaires indésirables. Avec l’objectif principal d’identifier des cibles pour des médicaments alternatifs, le génome nucléaire d’*E. multilocularis* et d’*E. granulosus* a été récemment séquencé, et de nombreuses cibles potentielles pour des médicaments sont exprimées par le métacestode. De plus, des études récentes de biologie cellulaire ont montré qu’*E. multilocularis* dispose de cellules souches multipotentes, appelées cellules germinales, qui sont les seules cellules parasitaires capables de prolifération et à l’origine de toutes les cellules différenciées. Ces cellules germinales représentent donc un type cellulaire crucial pour la prolifération d’*E. multilocularis*, et très vraisemblablement aussi d’*E. granulosus*, dans les organes de l’hôte, et vraisemblablement responsables des récurrences parasitaires à l’arrêt de la chimiothérapie. Des études récentes ont aussi indiqué que les cellules germinales pourraient être moins sensibles à la chimiothérapie car elles expriment un isoforme de la bêta-tubuline à affinité limitée vis-à-vis des benzimidazoles. Dans cet article, nous faisons une courte revue des découvertes récentes concernant la génomique d’*Echinococcus* et la recherche sur les cellules souches. Nous proposons que les recherches futures sur de nouveaux médicaments contre les échinococcoses se focalisent sur la population des cellules souches du parasite.

Cystic echinococcosis (CE) and alveolar echinococcosis (AE) are potentially lethal diseases that are caused by the metacestode larval stages of the tapeworms *Echinococcus granulosus* and *E. multilocularis*, respectively (for a comprehensive review of the complex *Echinococcus* life cycles, please see Eckert and Deplazes [3]). In both cases, infection of humans occurs through oral uptake of infective eggs that contain the oncosphere larva. Upon hatching in the intestine and penetration of the intestinal wall, the oncospheres undergo a metamorphosis toward the metacestode in the inner organs of the intermediate host, mostly affecting the liver. Particularly AE is very difficult to treat since the *E. multilocularis* metacestode grows infiltratively, like a malignant tumor, into the surrounding host tissue and even forms metastases in secondary organs at late stages of the disease [2, 10]. In most cases, AE is diagnosed too late to allow complete surgical resection of the parasite tissue, leaving chemotherapy as the only remaining treatment option [2, 10]. Current anti-AE chemotherapy relies on benzimidazoles (e.g. albendazole, mebendazole) which target parasite β-tubulin, thus preventing proper assembly of the cytoskeleton [1, 2]. Since its introduction in 1978, benzimidazole-based chemotherapy has significantly improved the life expectancy and prognosis of AE patients [2, 10]. However, due to the fact that parasite and host β-tubulin are highly similar [1], benzimidazole administration is associated with adverse side effects, is parasitostatic only and, as a consequence, often has to be applied life-long [2, 10]. Thus, particularly for the treatment of AE, novel chemotherapeutic options are urgently needed. In principle, this also holds true for CE although due to the fact that the *E. granulosus* metacestode grows non-infiltratively as a single (hydatid) cyst, CE is more accessible for surgery and shows slightly better responses to benzimidazole chemotherapy [2].

The quest for novel drug targets against echinococcosis, but also interest in a deeper understanding of host-parasite interaction and parasite developmental mechanisms, has for several years fuelled initiatives to characterize the whole nuclear genomes of *E. multilocularis* and *E. granulosus*, which culminated in the release of two highly recognized publications in 2013 [25, 26]. While Tsai et al. [25] reported the whole genome sequences of four tapeworms (*E. multilocularis*, several European isolates; *E. granulosus*, G1 strain, Uruguay; *Taenia solium*, Mexican isolate; *Hymenolepis microstoma*, Nottingham strain), with that of *E. multilocularis* as a high-quality reference genome, Zheng et al. [26] characterized the genome of a Chinese *E. granulosus* G1 isolate and, like Tsai et al. [25], supported their data by including comprehensive transcriptomic analyses of several parasite developmental stages. Both studies revealed extensive adaptations to parasitism in the tapeworm genomes such as the loss of several pathways important for the *de novo* synthesis of amino acids, nucleotides, fatty acids, and cholesterol, which have to be taken up from the host [25, 26]. Genes and gene families for the uptake of these nutrients, on the other hand, were either highly expressed in the metacestode stage, greatly expanded, or even newly evolved in cestodes [25, 26]. Likewise, cestodes appear to have expanded or evolved genes (mostly antigen-encoding) for the modulation of the host immune system [25, 26]. Importantly, both studies also identified promising drug targets such as G-protein-coupled receptors (GPCRs), ion channels, proteases and kinases that are expressed in the clinically relevant metacestode stage [25, 26] and against which lead substances are available that can be tested for antiparasitic activities in established *in vitro* [8, 20–22] and *in vivo* [14] models for *Echinococcus* infections. Most interestingly, these genomic analyses also yielded clear indications that cestodes, like the related flukes, apparently employ a highly modified stem cell system [25, 26]. Factors like Vasa (a classical germ cell marker in metazoans) and Piwi which are involved in maintaining pluripotency of germline cells in all Bilateria investigated so far, as well as multipotency in somatic stem cells of many invertebrate lineages (as part of the germline multipotency program, GMP), are obviously missing in the genomes of tapeworms [19, 25, 26]. Although the implications of these modifications on stem cell maintenance and dynamics in cestodes are not yet clear [19], they could be related to the unlimited proliferation capacity (literal “immortality”) typically observed in cestode larvae (e.g. *Echinococcus*) or adults (e.g. *Taenia*). Having been somehow “forgotten” by *Echinococcus* molecular and cellular research since their original (mostly morphological) description in the 1970s and 1980s [4, 15, 17, 24], these findings brought the *Echinococcus* stem cell population (called the “undifferentiated” or “germinative cells”) back into the focus of interest.

A necessary prerequisite for functional investigations into *Echinococcus* stem cells was the development of an axenic (host cell-free) *in vitro* cultivation method for *E. multilocularis* metacestode vesicles by Spiliotis et al. [22], followed by the establishment of the first culture system for parasite “primary cells” [20, 21]. Using these techniques, Koziol et al. [13] recently carried out an exhaustive analysis of stem cell proliferation and dynamics in *E. multilocularis* larvae. In particular, these authors developed methods to identify and track parasite germinative cells and differentiated cells of the metacestode (e.g. muscle cells, glycogen storage cells, tegumental cells) during the development and growth of parasite larvae [13]. As a differentiated cell type of special interest, they also included nerve cells which have just recently (and unexpectedly) been shown to be present as a “nerve net” in the metacestode [12]. The investigations of Koziol et al. [13] conclusively demonstrated that the germinative cells are the only cells in the *E. multilocularis* metacestode that are capable of proliferating, and that all differentiated cells of the metacestode originate from germinative cells. These investigations also revealed that *E. multilocularis* germinative cells not only differ from stem cells of other metazoans in the lack of Piwi and Vasa, but also from stem cell systems of other flatworms (planarians, schistosomes) in the expression patterns of additional GMP components such as *nanos* [13]. During the evolution of parasitism, the *E. multilocularis* germinative cells have thus been modified into a very unique stem cell system, which might be associated with the extensive asexual proliferation capacity within the host. Importantly, Koziol et al. [13] also developed methods to specifically eliminate stem cells in metacestode vesicles by hydroxyurea (HU) treatment, which had no effects on differentiated cells of the parasite. Although deprived of stem cells and, thus, of proliferation capacity, the treated vesicles remained structurally intact and survived for weeks in culture [13]. These techniques now provide access to the parasite’s germinative cell-specific transcriptome by simply subtracting the transcriptome of HU-treated vesicles from that of normal vesicles [13].

Since the germinative cells are the only proliferating cell type in *Echinococcus*, they must be the crucial drivers of the frequently observed recurrences [2, 10] of parasite development upon discontinuation of benzimidazole chemotherapy. Hence, benzimidazoles are obviously ineffective in killing the parasite’s stem cell population during chemotherapy. This is supported by *in vitro* studies [9, 23] showing that benzimidazole treatment of metacestode vesicles has very delayed effects on *E. multilocularis* “undifferentiated cells” (as called in these studies), which are actually the germinative cell population. Furthermore, at least in our hands, benzimidazole treatment does not show killing effects when applied to primary cell cultures after 2 days of development [18], which are highly enriched in germinative cells [13]. The most likely reason for benzimidazole insensitivity of germinative cells is that they may specifically express a β-tubulin isoform with limited affinity to benzimidazoles (as discussed by Schubert et al. [18]). As has been demonstrated by Brehm et al. [1], and verified through genomic and transcriptomic analyses [25], *E. multilocularis* expresses three major β-tubulin isoforms, called Tub-1, Tub-2, and Tub-3, which in almost identical form (99% identical amino acids) are also present in *E. granulosus* [25, 26]. While Tub-1 and Tub-3 contain amino acid motifs indicative of high affinity to benzimidazoles (e.g. phenylalanine at position 200), Tub-2 (with tyrosine at position 200) is most likely as insensitive to benzimidazoles as host β-tubulins (see discussion in Brehm et al. [1]). The recent transcriptomic analyses carried out for *E. multilocularis* showed that the Tub-2 encoding gene, *tub*-2, is by far the most highly expressed β-tubulin gene in *E. multilocularis* larval stages [16]. Furthermore, it displays a transcription profile (e.g. highest expression in primary cells at day 2 of development) that clearly suggests germinative cell-specific expression (discussed in Schubert et al. [18]). Taken together, these analyses indicate that the most important cell type for *Echinococcus* proliferation within the host, the germinative cells, is largely insensitive to current chemotherapy because it exclusively expresses a β-tubulin with limited affinity to benzimidazoles [18].

As a consequence of these findings, we suggest that future approaches of drug design and development against echinococcosis should also target the parasite’s stem cell system. Promising targets do not have to be specifically expressed in germinative cells, but they should also be expressed in these cells. In a recent contribution by Schubert et al. [18], for example, the *E. multilocularis* Polo-like kinase 1 (EmPlk1) has been targeted using the Plk-inhibitor BI-2536. Polo-like kinase 1 is an important cell cycle regulator necessary for undergoing mitosis and, accordingly, EmPlk1 is specifically expressed in germinative cells [18]. Interestingly, BI-2536 treatment had similar effects on *E. multilocularis* metacestode vesicles as HU treatment. The metacestode vesicles were specifically deprived of stem cells, ceased growth and proliferation, but remained intact for several weeks *in vitro* [18]. However, in contrast to HU, BI-2536 exerted its effects at concentrations (25 nM) that are much more relevant for clinical applications [18]. As a drug that specifically eliminates germinative cells, BI-2536 (or related, more parasite-specific compounds) could be ideal for complementing benzimidazole treatment which, when given alone, most probably only affects differentiated *E. multilocularis* cells. Other lead compounds that were already tested by us in *in vitro* systems for primary cells and metacestode vesicles were pyridinyl imidazoles, directed against p38 mitogen-activated protein kinase (MAPK) [5], Imatinib ([6] directed against Abl kinases), and HNMPA(AM)_3_ ([7]; insulin receptor kinases). All these compounds affected both metacestode vesicle development from primary cells and the integrity and survival of mature metacestode vesicles [5–7], although in the case of HNMPA(AM)_3_ very high doses of the drug had to be applied to achieve vesicle inactivation [7]. Interestingly, in transcriptomic analyses [25] the respective drug targets EmMPK2 [5], EmAbl1, EmAbl2 [6], and the insulin receptor-like kinases EmIR1 [11] and EmIR2 [7] turned out to be expressed by both metacestode vesicles and primary cell preparations that are enriched in germinative cells. This indicates that, at least *in vitro*, parasite development and survival can comprehensively be affected by drugs directed against a single target that is expressed by both germinative and differentiated *Echinococcus* cells, or by compounds that target several parasite factors of which at least one is expressed in germinative cells. None of the lead drugs mentioned above has yet been tested in an *in vivo* model of AE and it is indeed expected that this would be associated with adverse side effects since these compounds were originally developed and optimized against the orthologous mammalian kinases. However, the *in vitro* studies employing parasite germinative cells clearly demonstrate that the targeted kinases play an important role in parasite stem cell maintenance and/or differentiation, which opens the way for the development of more specific inhibitors of the parasite kinases that should cause few side effects.

In conclusion, genomic and transcriptomic analyses now provide for the first time a possible explanation for the high recurrence rates in AE chemotherapy: insensitivity of the parasite’s germinative cell population toward benzimidazoles due to the specific expression of a benzimidazole-resistant β-tubulin isoform in these cells. Future efforts of targeted drug design and development should thus concentrate on targets which are expressed in germinative cells since this is the crucial cell type driving parasite proliferation, differentiation, and regeneration within the host. The identification of respective drug targets will be greatly facilitated by analyses of the germinative cell-specific transcriptome through gene expression patterns obtained for parasite primary cell cultures (highly enriched in germinative cells) and hydroxyurea or BI-2536 treated metacestode vesicles [13, 18].
